# Evaluation of Comprehensive COVID-19 Testing Program Outcomes in a US Dental Clinical Care Academic Setting

**DOI:** 10.1001/jamanetworkopen.2022.46530

**Published:** 2022-12-13

**Authors:** Sung Eun Choi, Corneliu Sima, Laura Pesquera Colom, Giang T. Nguyen, William V. Giannobile

**Affiliations:** 1 Department of Oral Health Policy and Epidemiology, Harvard School of Dental Medicine, Boston, Massachusetts; 2 Department of Oral Medicine, Infection, and Immunity, Harvard School of Dental Medicine, Boston, Massachusetts; 3 Harvard University Health Services, Cambridge, Massachusetts

## Abstract

**Importance:**

Although many academic institutions have implemented infection control and prevention protocols, including regular asymptomatic self-testing, in response to the COVID-19 pandemic, the outcomes of mandatory surveillance testing programs at academic dental institutions that offer direct patient-facing clinical care has not yet been reported.

**Objective:**

To report the findings of a comprehensive surveillance COVID-19 testing program at an academic dental institution by assessing SARS-CoV-2 positivity rates and the potential association of test positivity with individual-level characteristics such as age, sex, and role.

**Design, Setting, and Participants:**

A retrospective cohort study was conducted using SARS-CoV-2 self-testing data from a mandatory surveillance program at the Harvard School of Dental Medicine. Test results obtained between August 24, 2020, and February 28, 2022, from students, faculty, and staff members were analyzed. Testing cadence varied from 1 to 3 times per week depending on risk status. The association of individual characteristics with test positivity was evaluated with univariate analyses and a bayesian multilevel logistic regression model.

**Exposures:**

Age by decade, sex, and role or position category (staff members, faculty, and students stratified by their involvement in clinical care activities), testing cadence, and testing date.

**Main Outcomes and Measures:**

Positive results from SARS-CoV-2 real-time reverse transcription–polymerase chain reaction self-tests were assessed.

**Results:**

Of the 390 study participants, 210 (53.8%) were women. Participants were grouped by age as follows: 20 to 29 years (190 [48.7%]), 30 to 39 years (88 [22.6%]), 40 to 49 years (44 [11.3%]), 50 to 59 years (42 [10.8%]), and 60 years or older (26 [6.7%]). Test results demonstrated an overall 0.27% positivity rate (61 test-positive cases), with a peak weekly positivity rate of 5.12% in the first week of January 2022. The mean (SD) test positivity rate among those involved in clinical activities was 0.25% (0.04) compared with 0.36% (0.09) among nonclinical participants. When adjusting for all considered covariates, test positivity was significantly associated with testing frequency (3 times vs 1 time per week: odds ratio [OR], 1.51 [95% credible interval (CrI), 1.07-3.69]) and timing of the test (after vs during the Alpha wave: OR, 0.33 [95% CrI, 0.11-0.88]; and Omicron vs Alpha: OR, 11.59 [95% CrI, 6.49-22.21]) but not with individual characteristics (age, sex, and role).

**Conclusions and Relevance:**

These findings suggest that implementing an adaptive testing cadence based on the risk status of individuals may be effective in reducing the risk of SARS-CoV-2 infection within an institution. In this study, involvement in clinical activities did not pose additional risk of SARS-CoV-2 infection compared with other in-person activities in the presence of these control measures.

## Key Points

**Question:**

Are individual characteristics associated with SARS-CoV-2 test positivity rates of a comprehensive mandatory surveillance COVID-19 testing program in a US dental clinical care academic setting?

**Findings:**

In this cohort study with 390 participants in clinical and nonclinical roles, the overall test positivity rate was 0.27%; the mean test positivity rate was 0.25% among those involved in patient-facing clinical activities compared with 0.36% among nonclinical participants. Test positivity was significantly associated with testing cadence but not with individual characteristics such as age, sex, and role.

**Meaning:**

These findings suggest that involvement in patient-facing dental clinical activities did not pose additional risk of SARS-CoV-2 infection compared with other in-person activities in the presence of intensive control measures.

## Teaser

This cohort study evaluates outcomes of a comprehensive mandatory COVID-19 surveillance testing program using individual characteristics associated with SARS-CoV-2 test positivity rates in a US dental clinical care academic setting.

## Introduction

The 2020 declaration of COVID-19 as a pandemic resulted in a global upheaval that caused the closure of businesses and academic institutions. In dental medicine, SARS-CoV-2 infection is a major concern because of the clinician’s close proximity to the patient’s oral cavity and the production of aerosols and droplets during dental procedures.^1,2,3^ The pandemic disrupted not only clinical care but also the didactic and research components of academic dental institutions. Although didactic content could be taught remotely using online platforms, preclinical activities requiring manikins were delayed. Research activities were also halted, resulting in great time and financial loss.^4,5^

In March 2020, Meng et al^6^ introduced essential knowledge about COVID-19 in dental care settings and provided recommendations for both dental practices and education. Since then, academic dental institutions have moved forward to reestablish in-person activities as understanding of SARS-CoV-2 transmission and testing continues to improve. Because of the high percentage of asymptomatic SARS-CoV-2 infections and their potential transmission risk,^7^ dental schools have applied public and local government guidelines, improved accessibility, assessed testing feasibility, and considered current epidemiological data to ensure faculty, staff, student, and patient safety. Furthermore, guidance on reopening institutions of higher education has helped mitigate the spread of COVID-19 on campus.^8,9^

Many institutions have incorporated reverse transcription–polymerase chain reaction (RT-PCR) testing at varying frequencies for asymptomatic surveillance of their community. Surveillance testing within institutions can be used as a tool in controlling the spread of COVID-19.^10,11,12^ In addition, surveillance testing enables early identification of individuals who may or may not be symptomatic and helps prevent the spread of disease via contact tracing and isolation.^13,14^

The Harvard School of Dental Medicine (HSDM), the only school at Harvard University that offers direct patient care within university-operated facilities, used surveillance testing and contact tracing to safely reopen when in-person activities resumed in fall 2020. In this study, we present the results of Harvard’s comprehensive, mandatory surveillance testing program by assessing HSDM positivity rates and the potential association of test positivity with individual-level characteristics (age, sex, and role), using deidentified data collected from 2020 through the beginning of 2022.

## Methods

The Harvard Medical School Institutional Review Board deemed this cohort study exempt from review because it did not constitute human participant research; informed consent was thus waived. The study followed the Strengthening the Reporting of Observational Studies in Epidemiology (STROBE) reporting guideline.

### Data Source and Study Population

**Figure 1.**
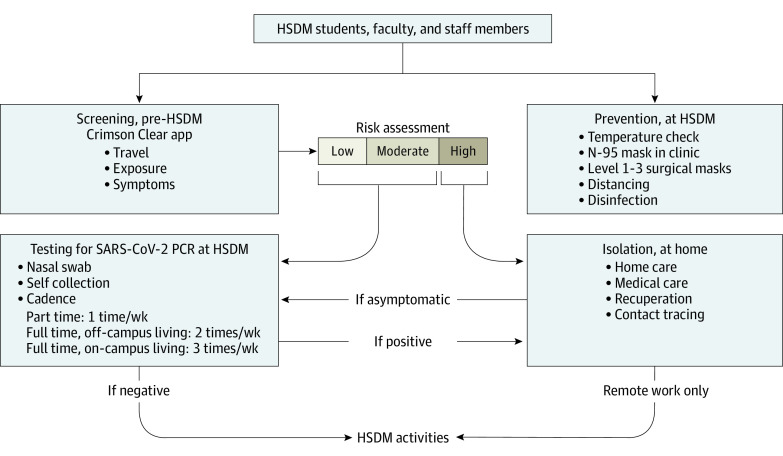
Study Design HSDM indicates Harvard School of Dental Medicine; PCR, polymerase chain reaction.

Beginning in August 2020, recurring COVID-19 testing was required for all individuals authorized to live in dormitory-style, on-campus housing or to regularly work on campus at HSDM. Participants who submitted their test results to the surveillance program included students (predoctoral, graduate, and research), faculty, and staff members (Figure 1). Participant testing cadence varied from 1 to 3 times weekly, depending on the regularity of their presence on campus, vaccination status, and on-campus residential status. Individuals who were incompletely vaccinated were required to test more frequently, as were those who lived in on-campus residence halls. Those who were up to date on vaccination and who lived off campus had the lowest testing frequency. Individuals who worked remotely and did not attend campus consistently were not required to enter the campus for the sole purpose of testing. Those with close contacts to positive cases were advised to test more frequently during the subsequent week; if these individuals had a positive test result, contact tracing was performed. Before the major surge in cases associated with the Omicron variant, personalized outreach was practiced by a dedicated team of registered nurses who were trained in contact tracing at Harvard University Health Services. Personalized outreach included email and phone contact and followed best practices informed by the US Centers for Disease Control and Prevention (CDC) and state public health guidance.^15,16^ When the volume of positive cases increased dramatically with Omicron, contact tracing focused largely on conducting automated outreach and addressing complex cases, whereas notification of close contacts became the responsibility of individuals with SARS-CoV-2 infection.

Results of real-time RT-PCR self-tests for SARS-CoV-2 infection were obtained from 391 individuals participating in the mandatory surveillance program at HSDM from August 24, 2020, through February 28, 2022. During the study period, 22 766 tests were processed; the results were obtained from the Broad Institute of MIT and Harvard and from the Harvard University Clinical Laboratory. Four records from 1 participant with failed or inconclusive test results were excluded from the analysis, resulting in 22 762 testing records from the 390 participants included in the study.

### Statistical Analysis

Variables collected and derived for participant characteristics included age group by decade (20-29, 30-39, 40-49, 50-59, and ≥60 years), sex, role or position category (staff members, faculty, and students stratified by their involvement in clinical activities, referred to hereafter as role), testing frequency (number of tests completed per week), timing of the test (corresponding to different COVID-19 variant waves: August 2020 to February 2021 [Alpha], March 2021 to June 2021 [after Alpha], July 2021 to November 2021 [Delta], and December 2021 to February 2022 [Omicron]), and test date. For our exploratory analyses, test positivity rates in the studied cohort were measured as percentages of total testing records, using testing-level data. Test results that were canceled, failed, or inconclusive were excluded (547 of 22 762 records). If an individual tested positive multiple times in a 90-day period, only the first positive test was counted. We evaluated how positivity rates varied over time on a weekly basis.

To assess the crude association of individual characteristics (age, sex, and role) with test positivity, individual-level univariate analyses were conducted. For these analyses, results were aggregated to the individual level, with the outcome defined as having a positive test result at least once during the study period. The χ^2^ or Fisher exact test was used depending on the number of observations in each category.

Using testing-level data, a bayesian multilevel logistic regression model was estimated to assess the association of individual characteristics with test positivity, adjusting for all available covariates such as age group, sex, role, testing frequency, and timing of the test. Testing frequency was included as a proxy to adjust for COVID-19 risk status. Timing of the test was included to account for SARS-CoV-2 infection rates over time and for different participation rates in the testing program across the study period (eFigure 1 in Supplement 1). The bayesian multilevel model was chosen because it is used (1) to aid in model convergence without needing to provide domain-specific prior information and (2) to account for repeated measures among participants by including a random effect at the individual level (eMethods in Supplement 1). Unadjusted and adjusted odds ratios (ORs) for the covariates considered are reported with 95% credible intervals (CrIs). Statistical significance for individual-level analysis was based on 2-sided *P* values ≤.05. For bayesian regression models, uncertainty in our estimates was represented using 95% CrIs computed from Markov chain Monte Carlo sampling. All analyses were performed with R, version 3.6.1 (R Foundation for Statistical Computing).

## Results

Of the 390 participants, 210 (53.8%) were women and 180 (46.2%) were men. Participants were grouped by age decade as follows: 20 to 29 years (190 [48.7%]), 30 to 39 years (88 [22.6%]), 40 to 49 years (44 [11.3%]), 50 to 59 years (42 [10.8%]), and 60 years or older (26 [6.7%]). Deidentified SARS-CoV-2 self-test results from our study included 22 762 testing records obtained between August 2020 and February 2022. There were 299 (76.7%) and 91 (23.3%) participants involved in clinical and nonclinical activities, respectively (Table 1). Time of testing program entry varied during the study period; the number of participants who submitted testing samples peaked from January 2021 through May 2021, with the weekly number of participants varying from 249 to 299 (eFigure 1 in Supplement 1). The mean overall testing frequency was 1.32 times per week (95% CrI, 0.80-1.83 times per week; Table 1); among participants involved in clinical activities, the mean testing frequency was higher than for nonclinical participants, at 1.37 times per week compared with 1.15 times per week (eFigure 2 in Supplement 1). Students involved in patient care were tested most frequently, followed by clinical staff members.

**Table 1.** Study Population From August 2020 to February 2022

Characteristic	No. of participants (%)^a^						
	Total (N = 390)	Nonclinical (n = 91)			Clinical (n = 299)		
		Students (n = 25)	Faculty (n = 12)	Staff (n = 54)	Students (n = 214)	Faculty (n = 66)	Staff (n = 19)
Age, y							
20-29	190 (48.7)	7 (28.0)	0 (0.0)	9 (16.7)	170 (79.4)	3 (4.5)	1 (5.3)
30-39	88 (22.6)	18 (72.0)	0 (0.0)	10 (18.5)	42 (19.6)	13 (19.7)	5 (26.3)
40-49	44 (11.3)	0 (0.0)	4 (33.3)	17 (31.5)	2 (0.9)	19 (28.8)	2 (10.5)
50-59	42 (10.8)	0 (0.0)	4 (33.3)	12 (22.2)	0 (0.0)	17 (25.8)	9 (47.4)
≥60	26 (6.7)	0 (0.0)	4 (33.3)	6 (11.1)	0 (0.0)	14 (21.2)	2 (10.5)
Sex							
Male	180 (46.2)	17 (68.0)	5 (41.7)	21 (38.9)	92 (43.0)	41 (62.1)	4 (21.1)
Female	210 (53.8)	8 (32.0)	7 (58.3)	33 (61.1)	122 (57.0)	25 (37.9)	15 (78.9)
No. of tests per week, mean (95% CrI)	1.32 (0.80-1.83)	1.05 (0.88-1.22)	1.05 (0.77-1.33)	1.22 (0.75-1.68)	1.44 (0.96-1.91)	1.14 (0.77-1.51)	1.41 (1.10-1.72)
No. with a positive test result							
Ever	56 (14.4)	1 (4.0)	0 (0.0)	14 (25.9)	25 (11.7)	13 (19.7)	3 (15.8)
1 case	52 (13.3)	1 (4.0)	0 (0.0)	13 (24.1)	24 (11.2)	13 (19.7)	1 (5.3)
2 cases	3 (0.8)	0 (0.0)	0 (0.0)	1 (1.8)	1 (0.5)	0 (19.7)	1 (5.3)
3 cases	1 (0.3)	0 (0.0)	0 (0.0)	0 (0.0)	0 (0.0)	0 (0.0)	1 (5.3)

^a^ Percentages are reported as fractions of column totals.

**Figure 2.**
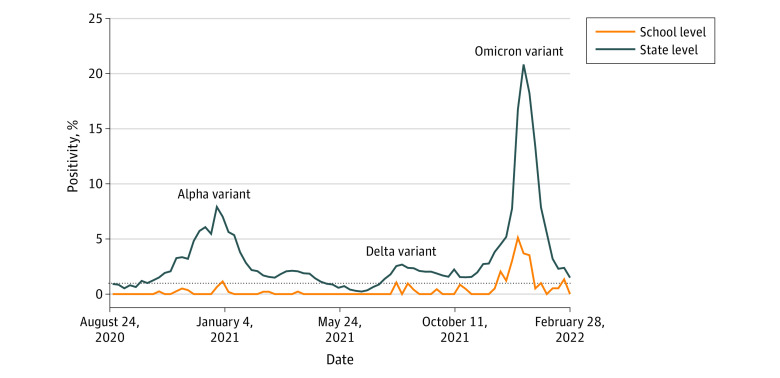
Comparison of SARS-CoV-2 Test Positivity Rates Among the Harvard School of Dental Medicine and Massachusetts Populations Weekly test positivity rates (for August 24, 2020, to February 28, 2022) were assessed. Alpha, Delta, and Omicron variant peaks are shown. Massachusetts data were obtained from the state COVID-19 interactive data dashboard.^17^

During the study period, the overall test positivity rate was 0.27% (61 test-positive cases; eFigure 3 in Supplement 1) and the mean (SD) weekly positivity rate was 0.36% (0.85). The mean (SD) test-positive rate among participants involved in clinical activities was 0.25% (0.04) compared with 0.36% (0.09) among nonclinical participants. The weekly test positivity rate peaked at 5.12% during the first week of January 2022, and the overall test positivity pattern was consistent with that observed in Massachusetts during different COVID-19 variant waves (Figure 2). At the individual level, 56 participants (14.4%) had at least 1 positive test result during the study period (Table 1) and 4 (1.0%) had more than 1 positive test result.

**Figure 3.**
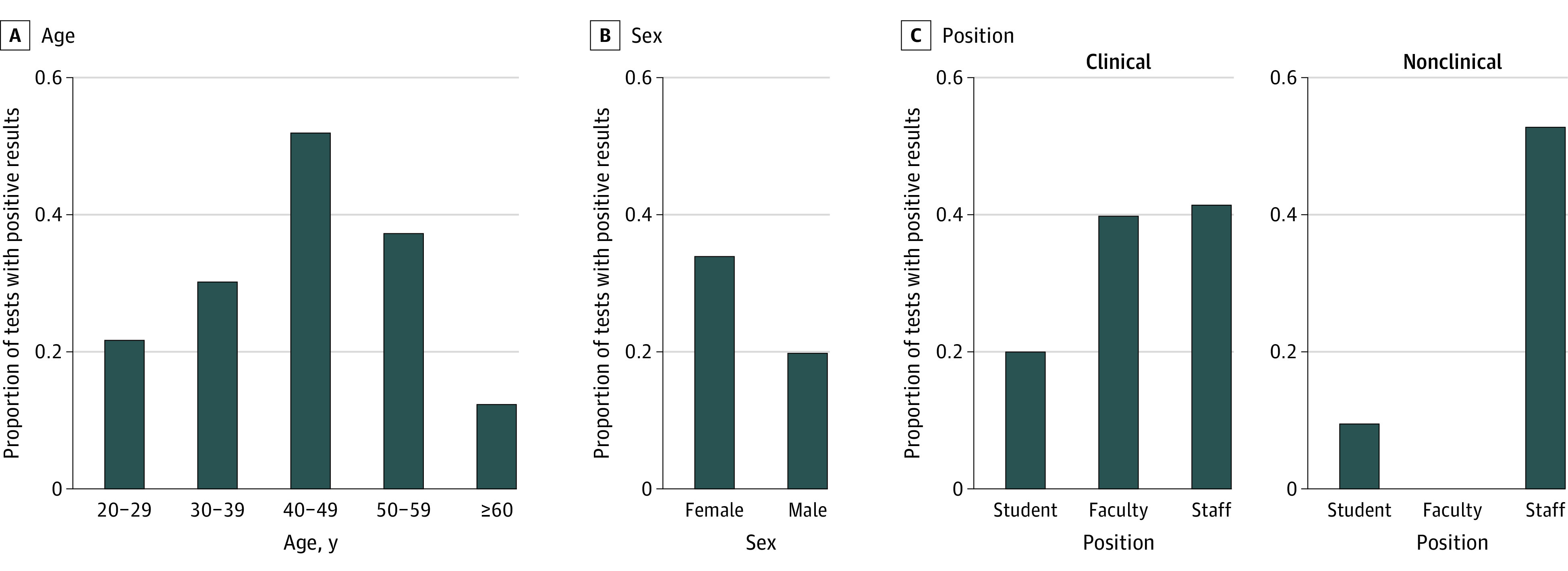
Percentage of SARS-CoV-2–Positive Test Results by Participant Characteristics, Including Age, Sex, and Role

Test positivity rates varied by individual characteristics. Based on our univariate analyses at the individual level, role was significantly associated with positive test results (eFigure 4 in Supplement 1). Nonclinical staff members tested most often, with a positivity rate of 0.53% during the study period (Figure 3). When adjusting for all covariates considered in the bayesian multilevel logistic regression model, test positivity was significantly associated with testing frequency (number of tests per week determined based on individual risk status) and timing of the test (corresponding to COVID-19 waves) using test-level data (Table 2). The likelihood of testing positive increased significantly for individuals required to test 3 times per week (OR, 1.51 [95% CrI, 1.07-3.69]) compared with testing only once per week. Our regression results also showed that compared with August 2020 to February 2021 (Alpha), the likelihood of testing positive decreased significantly between March 2021 and June 2021 (after Alpha; OR, 0.33 [95% CrI, 0.11-0.88]) and increased between December 2021 and February 2022 (Omicron; OR, 11.59 [95% CrI, 6.49-22.21]). When the model was fully adjusted, other individual characteristics (age group, sex, and role) were no longer associated with test positivity.

**Table 2.** Results of Bayesian Multilevel Logistic Regression Model

Characteristic	OR (95% CrI)	
	Unadjusted	Adjusted
Age, y		
20-29	1 [Reference]	1 [Reference]
30-39	1.58 (0.68-3.64)	1.27 (0.60-2.64)
40-49	3.02 (1.06-8.45)	1.28 (0.50-3.24)
50-59	1.73 (0.59-5.10)	0.85 (0.32-2.24)
≥60	0.59 (0.11-3.27)	0.43 (0.12-1.45)
Sex		
Female	1 [Reference]	1 [Reference]
Male	0.53 (0.27-1.06)	0.62 (0.34-1.14)
Role or position category		
Clinical		
Student	1 [Reference]	1 [Reference]
Faculty	1.72 (0.85-3.26)	1.72 (0.71-4.23)
Staff	1.97 (0.84-4.32)	2.01 (0.72-5.43)
Nonclinical		
Student	0.62 (0.20-1.69)	0.55 (0.18-1.58)
Faculty	0.49 (0.10-2.14)	0.50 (0.09-2.49)
Staff	2.03 (0.64-7.03)	2.12 (0.58-7.66)
No. of tests per week		
1	1 [Reference]	1 [Reference]
2	0.64 (0.33-1.28)	1.11 (0.62-1.91)
3	1.64 (1.21-5.64)^a^	1.51 (1.07-3.69)^a^
COVID-19 variant wave		
Alpha (August 2020 to February 2021)	1 [Reference]	1 [Reference]
After Alpha (March 2021 to June 2021)	0.22 (0.04-1.01)	0.33 (0.11-0.88)^a^
Delta (July 2021 to November 2021)	1.17 (0.47-2.93)	1.13 (0.47-2.54)
Omicron (December 2021 to February 2022)	15.35 (7.53-31.27)^a^	11.59 (6.49-22.21)^a^

Abbreviations: CrI, credible interval; OR, odds ratio.

^a^ Significant results with 95% CrIs not containing 1.

## Discussion

In this cohort study, the overall asymptomatic SARS-CoV-2 test positivity rate remained low at 0.27%. This finding suggests that the implemented infection control and prevention protocols were effective in reducing COVID-19 risk within a dental clinical care academic setting. We also observed that involvement in clinical activities did not seem to increase the risk of SARS-CoV-2 infection. Although individuals involved in clinical activities performed a higher number of tests per week on average, the test positivity rate remained lower than for nonclinical individuals, contributing to the safety of both patients and health care practitioners in clinical settings. In our adjusted regression model, individual characteristics were not significantly associated with positive test results, except for timing of the test and testing frequency, which was determined based on participants’ on-campus presence regularity, vaccination status, and on-campus residential status. This finding signifies the importance of adaptive testing cadence when implementing large-scale surveillance testing programs at academic institutions.

Because oral health care practitioners work in close proximity to patients’ mouth, nose, and throat, both patients and practitioners may be considered at high risk of SARS-CoV-2 infection. While dental care workers at HSDM used N95 masks and plexiglass shields, aerosol mitigation with aerosol-generating procedures, and physical distancing, the environment was considered safe and there were no documented cases of clinician-to-patient transmission (data not shown). Some dental procedures may lead to aerosol generation, further increasing transmission risk through direct inhalation or contact with contaminated surfaces.^18^ Because of the unique nature of dental care settings and interventions, the World Health Organization (WHO) initially advised in August 2020 that routine nonurgent oral health care be delayed until COVID-19 transmission rates were reduced from community transmission to cluster cases.^19^ To protect patients and the dental care team on resumption of dental care, the CDC, the American Dental Association, the US Occupational Safety and Health Administration, and the WHO released infection prevention and control guidelines for providing the full range of dental care.^20,21,22^ Safety of the dental office is a priority in dental clinical care academic settings. Implementation of comprehensive mandatory surveillance testing programs can help limit the spread of COVID-19 on campus when coupled with infection prevention and control guidelines. These programs also serve to create a sense of security for members of the academic, clinical, and patient care communities.^23^

In this study, the asymptomatic test positivity rate at HSDM was 0.27% among 390 students, faculty, and staff members from August 2020 through February 2022. However, a study of a SARS-CoV-2 surveillance testing program at the Georgia Institute of Technology (with 18 029 students, staff, and faculty) reported a mean asymptomatic test positivity rate of 0.84% during the 2020 fall semester.^24^ The Duke University surveillance program (with 10 265 students) reported a weekly per capita positivity rate of 0.08% during fall 2020.^25^ The results of surveillance testing programs at academic institutions varied widely, and these contrasting examples emphasize the need to assess the costs and benefits of implementing large-scale surveillance testing programs upon evaluating test sensitivity and retrospective serologic surveys.

We observed that higher testing frequency was associated with a higher test positivity rate. Testing frequency in our study was determined based on the risk status of individuals; to mitigate viral spread, a higher testing cadence was required for those at potentially increased risk of infection. This finding may seem contradictory to that of a previous modeling study that demonstrated no association between testing frequency and infection transmission.^26^ Although this hypothetical modeling study evaluated the association of varying testing frequencies applied universally with the overall infection rates as a means of rapidly detecting positive cases, it is important to note that our study is limited to the results of the asymptomatic mandatory testing program at Harvard, which cannot capture the overall positive cases detected from contact tracing and symptomatic monitoring. As such, we recognize that our findings specifically highlight the need for adaptive testing cadence based on the individual level of risk rather than assessment of the effectiveness of testing cadence on overall infection rates. Having access to complete data on SARS-CoV-2 infection status, which include the overall positive cases from contact tracing and symptomatic monitoring within the institution, would allow us to accurately evaluate the effectiveness of the mandatory surveillance testing program, which would inform quality improvement in the interest of institutional safety amid a public health emergency.

### Limitations

 This study has some limitations. As with any observational study, there may be unmeasured variables that confound the association of individual covariates with positive test results, such as other sociodemographic (race and ethnicity and educational attainment) and regional characteristics that were found to be substantial predictors of SARS-CoV-2 test positivity from this single center investigation.^27^ Additionally, because our data were collected from a single institution, the results may not be generalizable to other organizations implementing mandatory SARS-CoV-2 testing. Notwithstanding these limitations, the ability to better understand the interplay of a professional education program delivering clinical care and implications on COVID-19 transmission is a benefit of academic clinical settings.

## Conclusions

Implementation of adaptive testing cadence based on the risk status of individuals may assist with timely detection of SARS-CoV-2 infection and thus reduce the risk of infection within dental clinical care academic settings. In this study, dental clinical care activities, including aerosol-generating procedures, did not pose additional risk of SARS-CoV-2 infection compared with other in-person activities in the presence of these control measures. These findings suggest that the cumulative effect of these measures was successful in reducing the risk of infection associated with patient care.

## Notes

Supplement 1. **eMethods.** Bayesian Modeling **eFigure 1.** Number of Participants Submitting Testing Samples by Week **eFigure 2.** Distribution of Testing Frequency by Role or Position Categories **eFigure 3.** Characteristics of SARS-CoV-2 Testing Records **eFigure 4.** Univariate Analyses at the Individual Level

Supplement 2.
Data Sharing Statement
