# Supplementary material for: Evaluation of Comprehensive COVID-19 Testing Program Outcomes in a US Dental Clinical Care Academic Setting
Source: JAMA Netw Open. 2022 Dec 13;5(12):e2246530. doi: 10.1001/jamanetworkopen.2022.46530 (PMC9856527; doi:10.1001/jamanetworkopen.2022.46530)
Supplement: Supplement 1. — eMethods. Bayesian Modeling eFigure 1. Number of Participants Submitting Testing Samples by Week eFigure 2. Distribution of Testing Frequency by Role or Position Categories eFigure 3. Characteristics of SARS-CoV-2 Testing Records eFigure 4. Univariate Analyses at the Individual Level [file jamanetwopen-e2246530-s001.pdf]

Supplementary Online Content

Choi SE, Sima C, Pesquera Colom LP, Nguyen GT, Giannobile WV. Evaluation of comprehensive COVID-19 testing program outcomes in a US dental clinical care academic setting. *JAMA Netw Open*. 2022;5(12):e2246530. doi:10.1001/jamanetworkopen.2022.46530

eMethods. Bayesian Modeling

eFigure 1. Number of Participants Submitting Testing Samples by Week

eFigure 2. Distribution of Testing Frequency by Role or Position Categories

eFigure 3. Characteristics of SARS-CoV-2 Testing Records

eFigure 4. Univariate Analyses at the Individual Level

This supplementary material has been provided by the authors to give readers additional information about their work.

© 2022 Choi SE et al. *JAMA Network Open*

eMethods. Bayesian Modeling

A Bayesian method was chosen because of its improved convergence compared to the maximum likelihood method. Weakly informative prior distributions for all of the coefficients were specified as Normal (0, 1) which symbolizes neutral prior beliefs about the covariates' relationship with the outcome while simultaneously avoiding extreme odds ratio estimates. Priors for model intercepts were specified as Normal (0, 2.5) when the covariates are centered at their means. Priors for the random effect variance were specified as Gamma (1, 1). The NUTS sampler ran on 4 separate Monte Carlo Markov chains, each with 500 warmup iterations and 2000 sampling iterations. After the models were fit, we confirmed that the parameter estimates were stable by assessing the posterior density plots and trace plots as shown below. The patterns in the trace plots all appeared to be random and unassociated with previous values, and the density plots appeared roughly Normally distributed, implying that the parameter estimates had converged successfully.

Density and trace plots for the Bayesian regression model.

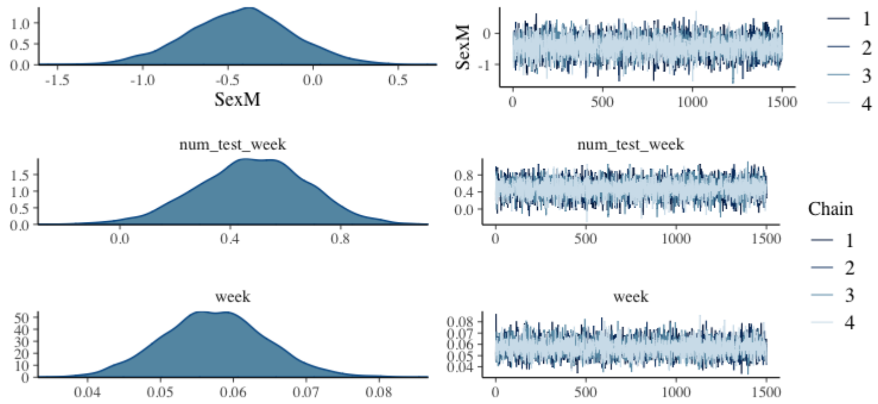

© 2022 Choi SE et al. *JAMA Network Open*

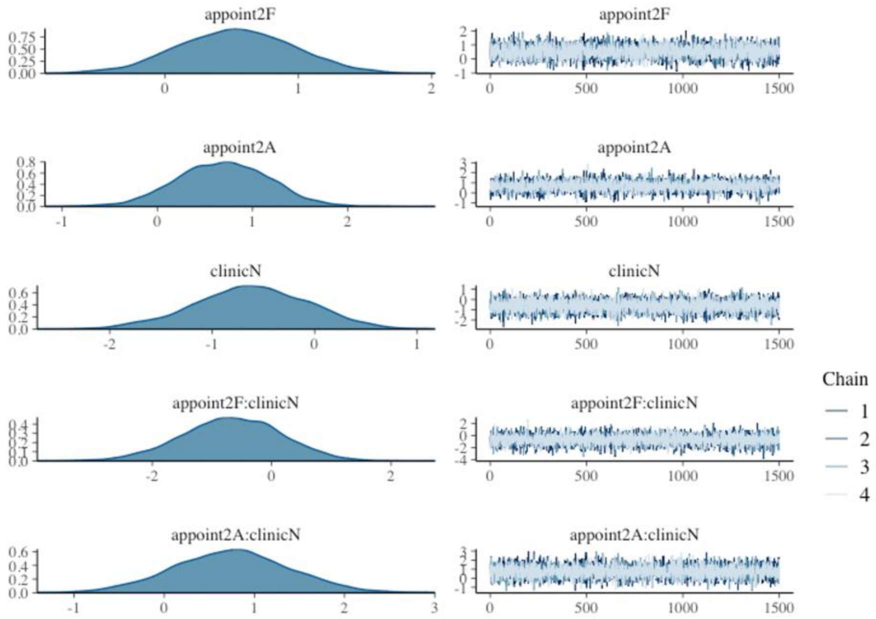

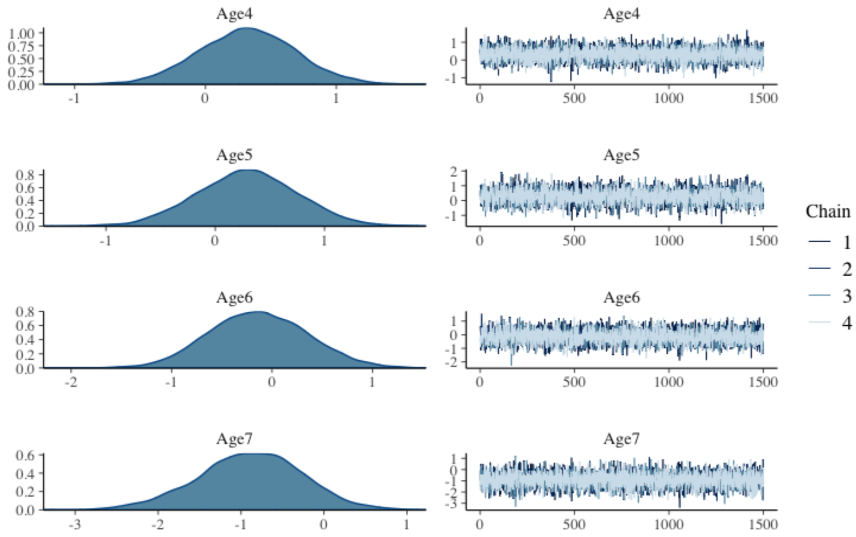

© 2022 Choi SE et al. *JAMA Network Open*

eFigure 1. Number of Participants Submitting Testing Samples by Week

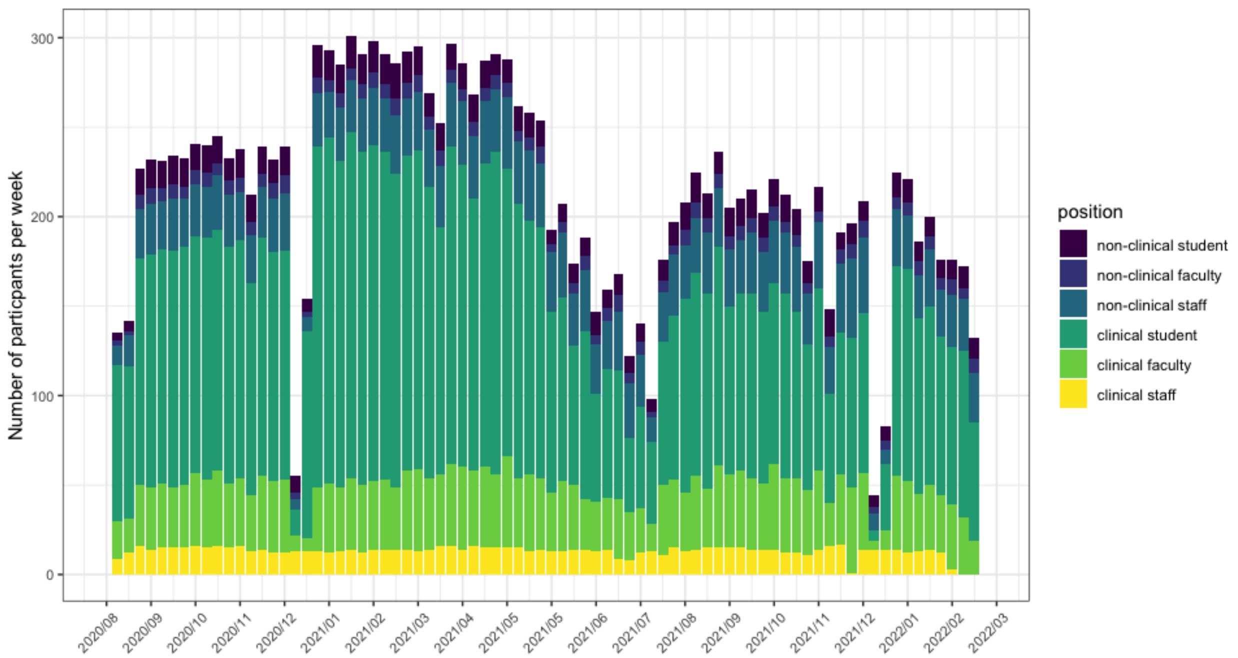

© 2022 Choi SE et al. *JAMA Network Open*

eFigure 2. Distribution of Testing Frequency by Role or Position Categories

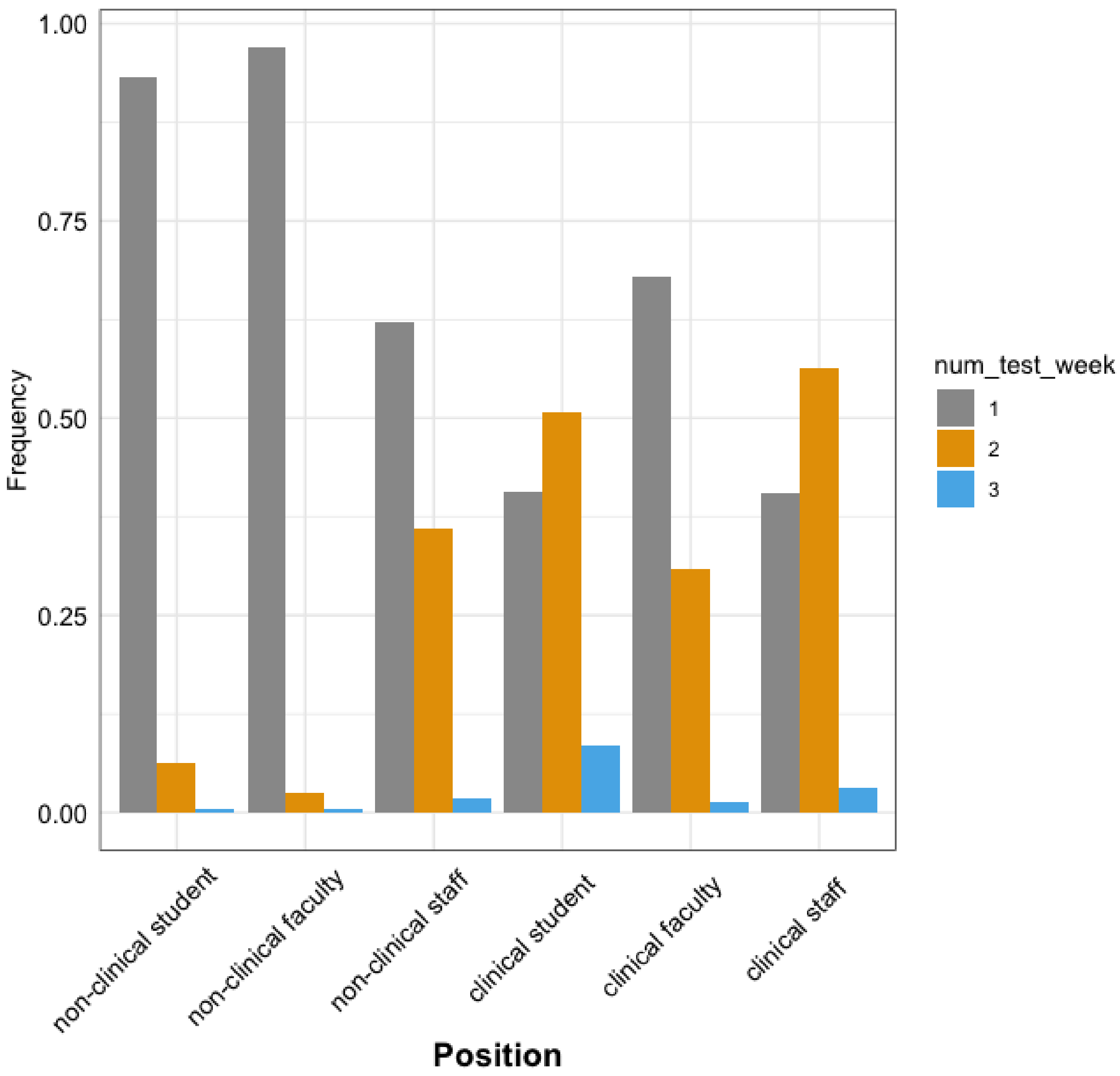

© 2022 Choi SE et al. *JAMA Network Open*

eFigure 3. Characteristics of SARS-CoV-2 Testing Records

Percentages of testing records by a) testing results, b) age group, c) sex, and d) position (role) category

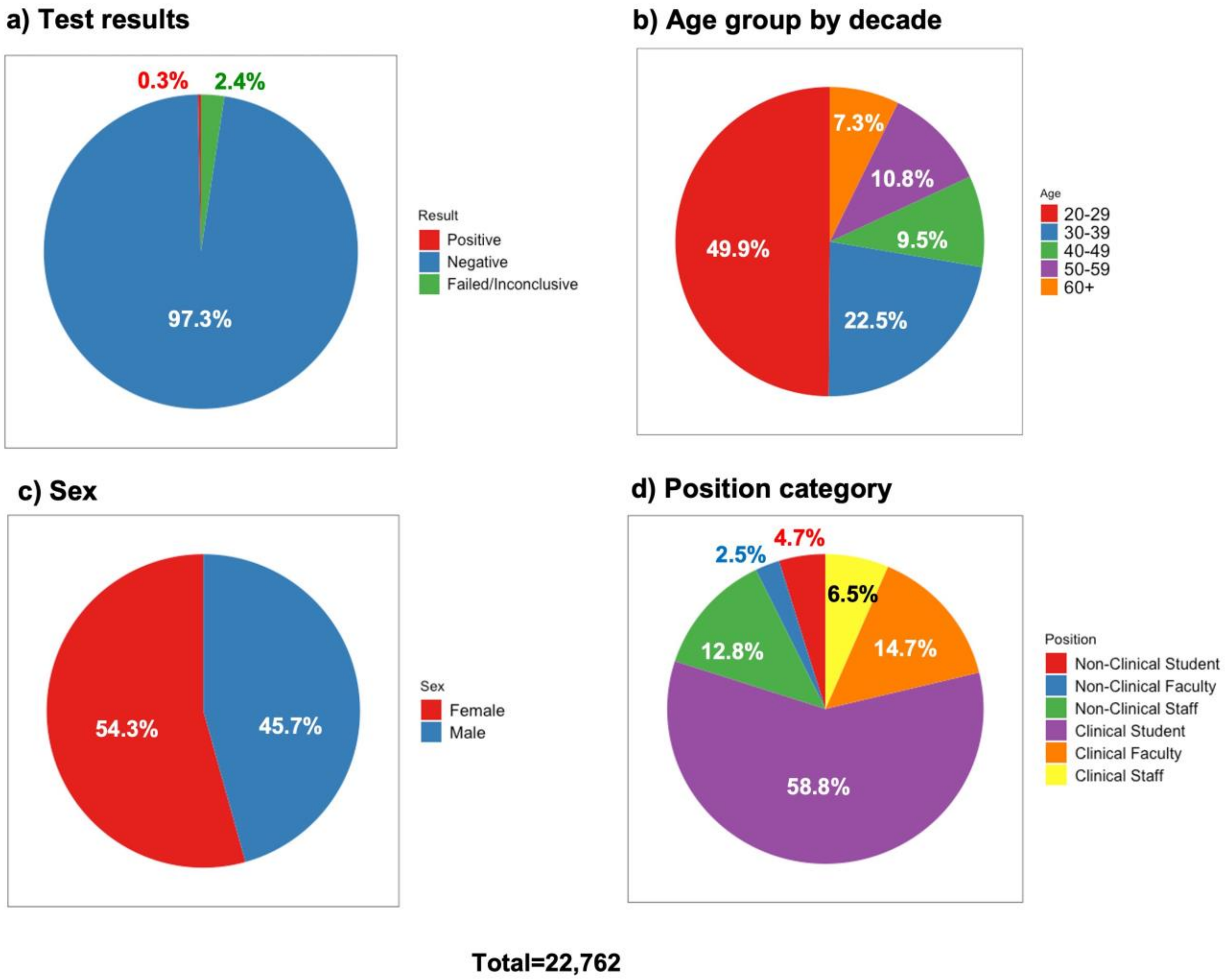

© 2022 Choi SE et al. *JAMA Network Open*

eFigure 4. Univariate Analyses at the Individual Level

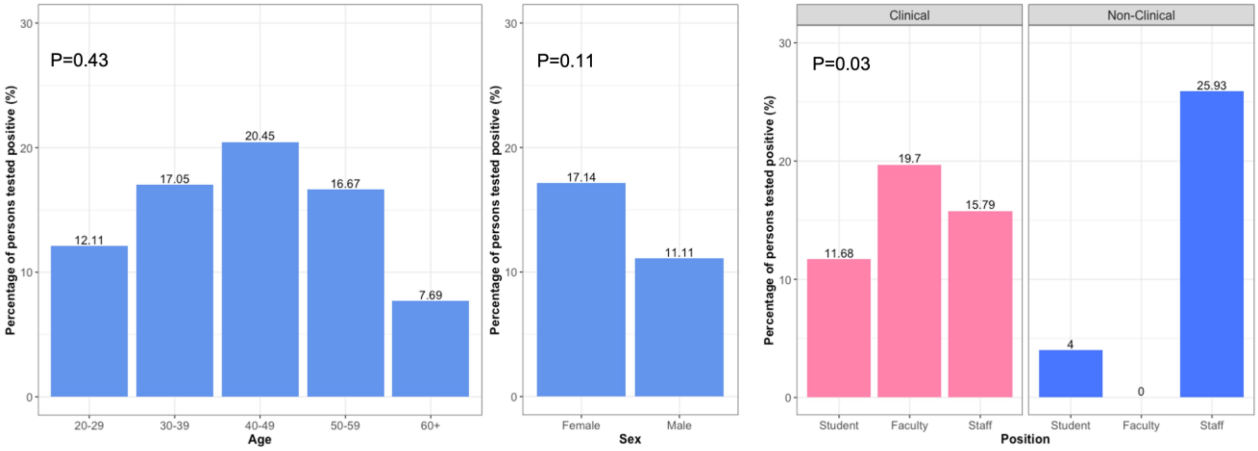

P-values are obtained from Chi-square or fisher's exact test was used depending on the number of observations in each category

© 2022 Choi SE et al. *JAMA Network Open*
