# Supplementary material for: Evaluation of Comprehensive COVID-19 Testing Program Outcomes in a US Dental Clinical Care Academic Setting
Source: JAMA Netw Open. 2022 Dec 13;5(12):e2246530. doi: 10.1001/jamanetworkopen.2022.46530 (PMC9856527; doi:10.1001/jamanetworkopen.2022.46530)
Supplement: Supplement 2. — Data Sharing Statement [file jamanetwopen-e2246530-s002.pdf]

Data Sharing Statement

Choi. Evaluation of Comprehensive COVID-19 Testing Program Outcomes in a US Dental Clinical Care Academic Setting. *JAMA Netw Open*. Published December 13, 2022. doi:10.1001/jamanetworkopen.2022.46530

Data
Data available: Due to privacy/ethical concerns, supporting data are not publicly available.
